# Prevalence and correlates of poor sleep quality in chronic liver disease patients

**DOI:** 10.5935/1984-0063.20200060

**Published:** 2021

**Authors:** Akash Kumar, Ravi Gupta, Rohit Gupta

**Affiliations:** 1 All India Institute of Medical Sciences, Psychiatry - Dehradun - Uttarakhand - India.; 2 All India Institute of Medical Sciences, Gastroenterology - Dehradun - Uttrakhand - India.

**Keywords:** Sleep, Depression, Restless Legs Syndrome

## Abstract

**Introduction:**

Previously done studies have shown that 39.6%-81% of subjects with chronic liver disease (CLD) report poor sleep quality and 42% experience insomnia. However, despite the high prevalence of insomnia and poor sleep quality in this group of patients, literature is scanty. In addition, previous studies have not ruled out subjects with restless legs syndrome, which is seen in a sizable number of subjects having CLD.

**Material and Methods:**

Adult patients with a clinical diagnosis of CLD were included after excluding potential confounders. The etiology of CLD was investigated. The severity of liver disease was assessed and graded as Child-Turcotte-Pugh (CTP) class A, B or C; model for end-stage liver disease (MELD) score and as the presence of compensated or decompensated liver disease. Acute on chronic liver failure was also deﬁned as per APASL criteria. For the present study, subjects having a score greater than 14 on insomnia severity index along with clinical diagnosis (DSM-5) were considered as having insomnia. Depression was diagnosed using a patient health questionnaire (PHQ-9) along with clinical criteria following DSM-5. Sleep quality was assessed by the Pittsburg sleep quality index - Hindi version. RLS was diagnosed on clinical interview and examination. The severity of RLS was assessed using international RLS severity rating scales.

**Results:**

This cross-sectional study included 131 subjects. This sample had a predominance of males (78.6%), the average age of subjects was 48.70+12.31 years and 98.5% of subjects had decompensated liver disease. 54.2% had a history of alcohol use disorder and 45% had a history of nicotine use disorder. The prevalence of hepatitis B and C infection was 16.8% and 23.7%, respectively. Acute on chronic liver failure was observed in 22.9% of subjects. 19.8% of subjects had acute kidney injury. Poor sleep quality was reported by 37.4% of subjects in this study which was higher than population prevalence (p<0.001). Subjects with poor sleep quality had a higher proportion of insomnia, RLS, and depression. 19.8% of subjects reported insomnia in the present study and depressive symptoms were more severe among subjects with insomnia. RLS was reported by 19.1% of subjects and 2.3% had a positive family history of RLS. However, there was no difference in sleep quality and insomnia in patients with or without RLS.

**Conclusion:**

The present study shows that insomnia and poor sleep quality are more prevalent among patients with CLD. Sleep disturbance is associated with depressive symptoms and can worsen the quality of life.

## INTRODUCTION

Insomnia disorder is characterized by recurrent complaints (>3 nights a week) lasting for at least three months and associated with an inability to fall asleep, recurrent awakenings, or awakening before the desired wake time along with daytime symptoms of fatigue, irritability, cognitive disturbances, and change in appetite^1^. However, quality of sleep is a subjective phenomenon and is defined by a combination of a feeling of restorative sleep upon awakening, feeling energetic during the day, and the number of awakenings during the night^2^. Attempts have made to correlate subjective sleep quality with polysomnographic variables, but research has shown that objective measures of sleep are unable to differentiate good versus poor sleep quality^3^.

Induction and maintenance of sleep is a complex physiological process that requires interaction between circadian rhythm and sleep-pressure, both of which are influenced by several factors like neuronal functioning, emotions, pain, and physiological parameters of other systems of the body^4^. Change in physiological functions, as seen in various disease conditions can give rise to insomnia and impair sleep quality^1,4^. Insomnia and poor sleep quality at night may mimic depression, may predispose for development of depression, or may be a symptom of underlying depression^5^.

Several studies have shown that chronic liver disease impairs subjective sleep quality. It has been reported that the poor sleep is seen in 39.6%-81%^6,7^and insomnia has been reported in 42% of subjects with chronic liver disease^8^. Literature shows that many factors are associated with poor quality sleep like hypoalbuminemia, opiate therapy, leg cramps, female gender, covert hepatic encephalopathy, and low hemoglobin^6,9^. None of these factors could be replicated in any of the studies and one of the studies did not find any clinical or laboratory correlate of poor sleep quality among subjects with CLD^7^. Similarly, insomnia has been correlated to hepatitis C, Child-Turcotte-Pugh Grade A, and old age^8^.

As already discussed, depression has a bilateral relationship with insomnia, yet this issue has been assessed only in a couple of studies among chronic liver disease subjects. It has been reported that nearly 50% of the subjects of CLD suffer from depression^8^. Interestingly, Al-Jahdali et al.^8^reported that depression was more frequent among subjects without insomnia, rather than with insomnia.

However, despite the high prevalence of insomnia, poor sleep quality, and depression in CLD patients, literature is scanty^6-9^. Also, previous studies have not ruled out subjects with restless legs syndrome which is seen in a sizable number of subjects with CLD. Second, most of these studies are silent about the status of withdrawal from addictive substances in the study population. Third, clinical and laboratory correlates of poor sleep quality are variable across studies. Lastly, the relationship between insomnia, poor sleep quality, and depression has been assessed only in one study^8^.

Hence, the authors hypothesized that the prevalence of quality of sleep among patients with CLD would be higher than the population prevalence. We also hypothesized that after excluding confounding factors, e.g., RLS and substance use, subjects having depression, and more severe liver disease would have poorer sleep quality.

## MATERIAL AND METHODS

This cross-sectional observational study was conducted after obtaining approval from the institutional ethics committee. All subjects presenting with complaints of chronic liver disease admitted in the indoor patient department of the gastroenterology unit were approached and explained the rationale of the study. The study was done following a convenient sampling method over a period of 1 year (February 2019 to February 2020). Written informed consent was taken from all the subjects.

Chronic liver disease (CLD) was defined as an ongoing hepatic injury that lasts for at least 6 months due to a variety of etiological causes and progress to cirrhosis and end-stage-liver-disease. It was diagnosed by a combination of history, clinical examination, biochemical investigations, and radiological findings.

Subjects of either gender, age more than 18 years were included in this study. Subjects with altered sensorium, pregnant women, regularly taking any of the following medications, e.g., anticonvulsants, alpha-2-delta ligands, dopamine agonists and antagonists, histamine antagonists, benzodiazepines, oral iron tablets, opioids, selective serotonin reuptake inhibitors, and serotonin-norepinephrine reuptake inhibitors were excluded from the study since these medications could influence study variables like sleep-quality, insomnia, and RLS. Subjects with cannabis use disorder and opioid use disorder in the past 6 months were also excluded from the study as these have been found to influence the prevalence of RLS^1,10^.

### Calculation of sample size

The sample size was calculated a priori considering the proportion of RLS, depression, insomnia, and poor sleep quality in the general population and among patients with CLD based on available literature. Power was set to 80% and alpha to 0.05. The sample was calculated considering the prevalence of RLS, depression, insomnia, and poor sleep quality among the general population as 2.5%, 5%, 4.7%, and 10%, respectively, and among patients with CLD as 12%, 30%, 42%, and 50%, respectively^11-14^. This gave a sample size of 127 for RLS, 33 for depression, and 17 for insomnia as well as poor sleep quality. Since all these factors were to be analyzed in this study, the sample size was taken at 127 with 5% non-responders. Finally, 131 subjects agreed to provide complete information and hence, were included in this study.

### Etiology of liver disease

All patients admitted in the gastroenterology in-patient department were evaluated for their etiology of chronic liver disease. Detailed history for ethanol consumption was taken and patients with a history of significant daily ethanol consumption (>40g/d in males and >20g/d in females) were labeled as ethanol associated liver disease. All patients underwent serological markers to identify other etiological factors of CLD. These include viral markers for hepatitis (HBsAg, anti-HCV); autoimmune hepatitis profile (ANA, anti-SMA, anti-LKM, AMA antibodies); Wilson disease (KF ring, serum ceruloplasmin levels). Patients were diagnosed to have non-alcoholic fatty liver diseases (NAFLD) associated CLD if they were obese or had lifetime body weight in the obesity range or had an underlying history of diabetes and excluding other causes of CLD. A specific etiological workup was done for other etiologies of CLD.

### Severity of chronic liver disease

The severity of liver disease was assessed and graded as Child-Turcotte-Pugh (CTP) class A, B or C; model for end-stage liver disease (MELD) score, and as the presence of compensated or decompensated liver disease^15,16^. Decompensated liver disease was defined using the Asian Pacific association for study of liver disease (APASL) criteria, i.e., presence, currently or in past, of any one or more of the following: ascites, hepatic encephalopathy, variceal bleeding, serum bilirubin (>2.5 times the upper limit of normal) and prolonged prothrombin time (prolonged by >3s or an international normalized ratio (INR) >1.5), or any combination of them^17^. Acute on chronic liver failure was also defined as per APASL criteria^18^.

### Diagnosis of RLS

The diagnosis of RLS was made following the international restless legs syndrome study group criteria^19^. For the diagnosis of RLS, patients were screened using RLS diagnostic index for the Indian population by one of the authors (AK)^20^. “RLS diagnostic index for Indian population” includes RLS mimics and has a positive predictive value of 87.8% and a negative predictive value of 75% when the comparison group included subjects with RLS mimics, somatic symptoms disorder, and anxiety^20^.

All cases were then independently clinically examined by another author (RG) to confirm the presence of RLS. Wherever the diagnosis was in doubt, such subjects were instructed to watch for leg-symptoms and were interviewed again.

Wherever reliable information was not available, subjects were excluded from the study. Care was taken to exclude RLS mimics as mentioned in diagnostic criteria as well as paresthesia and burning feet syndrome from RLS^19^.

### Assessment of insomnia

Insomnia was assessed using the Hindi version of the insomnia severity index (ISI)^21,22^. ISI contains seven items that enquire about nighttime sleep and daytime symptoms of insomnia. It has a score ranging between 0 to 28. It classifies subjects into four categories: “no or minimal insomnia”, “subthreshold insomnia”, “moderate insomnia”, and “severe insomnia”. For the present study, subjects having a score greater than 14 along with clinical diagnosis were considered as having insomnia^1,23^. Hindi version of the instrument has an internal consistency of 0.91^22^.

### Assessment of mood

Hindi version of the patient health questionnaire (PHQ-9) was used to assess mood in the present study^24^. It assesses subjects on nine criteria for depression according to diagnostic and statistical module - IV edition (DSM-IV) on a four-point Likert scale ranging from not at all (score 0) to nearly every day (score 3). PHQ score of 0-4 shows no or minimal depression. Scores between 5-9, 10-14, 15-19, and >20 represent mild, moderate, moderately severe, and severe depression, respectively. Depression was considered when the PHQ score was greater than 10 along with clinical diagnosis based on DSM-5^1,24^.

### Sleep quality

Sleep quality was assessed by the Pittsburg sleep quality index - Hindi version^25^. It has nineteen items that are scored for seven subscales. A combined score of seven components provides a total score. It assesses subjective sleep quality over the past month. The global score of five or more represents poor sleep quality. It has a sensitivity of 89.6% and specificity of 86.5% with the internal consistency of 0.75 to differentiate between good sleepers and poor sleepers^25^.

### Comorbid medical disorders

Comorbid medical disorders were diagnosed based upon available medical records, history, clinical examination, and laboratory investigations. All subjects were taking medications for co-morbid medical disorders and were having a stable disease at the time of recruitment. Acute kidney injury was defined as serum creatinine more than 1.5mg% for this study.

### Addiction

Nicotine use disorder and tobacco use disorder were diagnosed following standard definitions as per diagnostic and statistical methods 5 (DSM-5) criteria^1^. None of the subjects was experiencing withdrawal symptoms at the time of recruitment in the study and none of the patients were on any substitution therapy.

### Laboratory investigations

A morning fasting sample of blood was collected from the antecubital vein after taking all aseptic precautions. Samples were sent for assessment of hematological parameters, liver function test, kidney function test, serum iron, serum ferritin, markers of hepatitis B, hepatitis C, and HIV infection. They were also subjected to ultrasonography of the hepatobiliary system.

### Statistical analysis

The analysis was done using Statistical Package for Social Sciences (SPSS) version 24.0 (IBM Corp., released 2016, IBM SPSS Statistics for Windows, version 24.0, Armonk, NY: IBM Corp.). Descriptive statistics were calculated. Chi-square test or Fisher’s exact test, as applicable were used to assess the strength of association between categorical variables. Some of the etiological factors of CLD had a small number in each sub-category, hence categories were collapsed to make three major categories: alcohol, infection (hepatitis B and C), and others (Table 1).

**Table 1.** Comparison of subjects with and without poor sleep quality.

S.N.	Variable	Whole sample (N=131)			Sample after removing RLS (N=106)		
		Good sleep (n=82)	Poor sleep (n=49)	p	Good sleep (n=74)	Poor sleep (n=32)	p
1.	Gender Male	63 (76.8%)	40 (81.6%)	0.51	56 (75.7%)	27 (87.4%)	0.31
2.	Age (years) ^	50.15 + 12.65	46.28 + 11.45	0.17	50.21 + 12.81	47.71 + 10.7	0.33
4.	Depression	2 (2.4%)	17 (34.7%)	<0.001	1 (1.4%)	11 (34.4%)	<0.001
5.	Insomnia	1 (1.2%)	25 (51.0%)	<0.001	1 (1.4%)	15 (46.9%)	<0.001
6.	RLS	8 (9.8%)	17 (34.7%)	<0.001			
7.	Etiology Alcohol Infection Others	31 (37.8%)	20 (40.8%)	0.89	27 (36.5%)	15 (46.9%)	0.58
		23 (28%)	12 (24.5%)		21 (28.4%)	7 (21.9%)	
		28 (34.1%)	17 (34.7%)		26 (35.1%)	10 (31.2%)	
							
8.	CTP grade A B C	11 (13.4%)	6 (12.2%)	0.82	10 (13.5%)	3 (9.4%)	0.81
		29 (35.4%)	20 (40.8%)		27 (36.5%)	13 (40.6%)	
		42 (51.2%)	23 (46.9%)		37 (50.0%)	16 (50%)	
9.	Anemia #	2 (2.4 %)	6(12.2%)	0.06	2(2.7%)	3(9.3%)	0.32
10.	COPD #	3 (3.6%)	1 (2.04%)	0.99	3 (4.05%)	0 (0%)	0.55
11.	Chronic kidney disease #	0 (0%)	2 (4.085)	0.13	0 (0%)	2 (6.25%)	0.08
12.	Altitude >1000masl #	11 (13.4%)	2 (4.1%)	0.07	10 (13.5%)	2 (6.2%)	0.23
13.	Acute on chronic liver failure	19 (23.2%)	11 (22.4)	0.92	16 (21.6%)	8 (25%)	0.70

# Fisher’s exact test;

^ Mann-Whitney U test; rest-: Chi-Square test.

Distribution of normalcy of numerical variables was assessed using the Kolmogorov-Smirnov test. Statistical significance of proportions was compared using the N-1 Chi-squared test. The difference in means of normally distributed variables was compared using independent sample t-test and the statistical significance of numerical variables with skewed distribution was analyzed using the Mann-Whitney-U test. Bonferroni correction was done for multiple analyses. Hence, statistical significance was considered at the *p*-value of 0.004 for Table 1 and 0.002 for Table 2.

**Table 2.** Comparison of subjects with and without poor sleep quality.

S.N.	Variable	Whole sample (N=131)			After removing RLS (N=106)		
		Good sleep (n=82)	Poor sleep (n=49)	p	Good sleep (n=74)	Poor sleep (n=32)	p
	**Hematological**						
1.	Hemoglobin (gm %) #	9.93 + 2.49	9.05 + 2.22	0.04	10.04 + 2.43	8.93 + 1.9	0.02
2.	Total RBC (x 1000/mm3) #	3.35 + 0.89	3.14 + 0.77	0.15	3.40 + 0.88	3.06 + 0.64	0.05
3.	Hematocrit (%)#	31.01 + 7.68	28.65 + 6.69	0.07	31.32 + 7.59	28.14 + 5.93	0.03
4.	Mean corpuscular hemoglobin (pg/cell) #	29.66 + 4.01	28.87 + 4.36	0.29	29.62 + 4.15	29.10 + 4.07	0.55
5.	Mean corpuscular volume (liters/cell)	92.36 + 10.01	90.20 + 10.71	0.13	92.03 + 10.29	90.25 + 11.02	0.30
6.	MCHC (gm/L)	31.67 + 2.02	31.79 + 2.10	0.69	31.70 + 2.08	32.25 + 1.86	0.22
	**Iron metabolism**						
7.	Serum iron (microgram/dl)	61.64 + 34.92	62.63 + 41.20	0.90	61.56 + 36.03	76.09 + 39.98	0.09
8.	Serum ferritin (microgram/l)	375.13 + 420.52	430.71 + 482.28	0.88	391.91 + 436.86	436.97 + 415.90	0.24
	Liver function tests						
9.	Total protein (g/dl) #	6.55 + 1.16	6.64 + 1.47	0.69	6.52 + 1.1.7	6.46 + 1.38	0.80
10.	Serum albumin(g/dl) #	2.94 + 0.77	2.87 + 0.54	0.58	3.01 + 0.74	2.84 + 0.56	0.24
11.	Serum globulin(g/dl)	3.61 + 1.04	3.58 + 1.11	0.88	3.51 + 1.00	3.61 + 1.18	0.52
12.	Serum total bilirubin (mg/dl)	6.32 + 7.78	6.72 + 8.39	0.99	6.28 + 7.96	7.54 + 8.68	0.34
13.	Serum direct bilirubin (mg/dl)	3.51 + 4.46	4.48 + 7.38	0.85	3.49 + 4.54	5.37 + 8.45	0.38
14.	ALT (U/l)	67.13 +	59.40 +	0.03	69.36 + 116.14	64.97 +	0.03
		111.08	118.01			140.33	
15.	AST(U/l)	120.80 + 159.63	132.28 + 402.86	0.35	127.21 + 166.48	156.60 + 494.90	0.39
16.	Serum alkaline phosphatase (U/l)	301.18 + 234.88	257.59 + 186.53	0.24	305.76 + 243.80	261.89 + 175.27	0.50
17.	Serum GGT(U/l)	232.46 + 430.22	177.58 + 136.81	0.24	243.55 + 448.31	129.64 + 156.29	0.32
18.	Prothrombin time (sec)	19.18 + 6.40	18.97 + 9.82	0.46	19.13 + 5.93	19.51 + 11.54	0.72
19.	INR	1.66 + 0.54	1.75 + 0.98	0.74	1.65 + 0.52	1.84 + 1.15	0.92
	**Renal function tests**						
20.	Serum urea (mg/dl)	43.57 + 29.44	47.69 + 48.31	0.87	43.89 + 30.02	53.80 + 50.78	0.67
21.	Serum creatinine(mg/dl)	1.23 + 0.85	1.28 + 1.22	0.48	1.24 + 0.88	1.45 + 1.46	0.87
22.	MELD score #	21.64 + 7.47	22.44 + 8.02	0.56	21.48 + 7.52	24.12 + 8.12	0.12

# Independent sample t test; Rest are Mann-Whitney U test.

## RESULTS

This cross-sectional study included 131 subjects. This sample had a predominance of males (78.6%), the average age of subjects was 48.70+12.31 years and 98.5% of subjects had decompensated liver disease. 20.6% had diabetes mellitus, 6.9% had systemic hypertension, 1.5% had chronic kidney disease, 6.1% had anemia, 3.1% had chronic obstructive pulmonary disease. 54.2% had a history of alcohol use disorder and 45% had a history of nicotine use disorder. Prevalence of hepatitis B and C infection was 16.8% and 23.7%, respectively; however, none of the subjects had HIV infection. Acute on chronic liver failure was observed in 22.9% of subjects. 19.8% of subjects had acute kidney injury. RLS was reported by 19.1% subjects which were significantly greater than population prevalence (*p*<0.001; 95% CI for difference 10.7-24.2)^14^. 2.3% had a positive family history of RLS. Similarly, the prevalence of depression in the present study (14.5%) was also higher than population prevalence (*p*<0.001; 95% CI for difference 3.7-15.9)^12^.

### Sleep quality

Poor sleep quality was reported by 37.4% of subjects in this study. This was significantly higher than the population prevalence reported in the previous study (*p*<0.001; 95% CI for difference 19.7-36.3)^11^. Subjects with good and poor sleep quality were comparable with regards to diabetes mellitus (*p*=0.39), systemic hypertension (*p*=0.24), acute kidney injury (*p*=0.56), hepatitis B infection (*p*=0.28), and hepatitis C infection (*p*=0.49). Both groups were also comparable with regards to alcohol use disorder (*p*=0.60) and nicotine use disorder (*p*=0.14). Subjects with poor sleep quality had a higher proportion of insomnia, RLS, and depression (Table 1).

There was no difference between these groups with regards to most of the hematological and biochemical parameters (Table 2). Though a statistically significant difference was observed between subjects with good sleep quality and poor sleep quality with regards to hemoglobin and serum AST, this difference was clinically insignificant (Table 2).

### Insomnia

19.8% of subjects reported insomnia in the present study. This was higher than the population prevalence reported in the earlier study (*p*<0.001; 95% CI for difference 9.1-22.7)^13^. Subjects with and without insomnia were comparable with regards to the altitude of residence (*p*=0.06), gender (*p*=0.76), the history of alcohol (*p*=0.63) and tobacco use (*p*=0.45), acute on chronic liver failure (*p*=0.61), and acute kidney injury (*p*=0.31). These groups were comparable with regards to hematological and biochemical parameters (data not shown). However, depressive symptoms were more severe among subjects with insomnia.

### Removal of cases with RLS

RLS is associated with insomnia as well as poor sleep quality in the past^26,27^. Hence, sleep quality was analyzed after removing cases of RLS. Results remain unchanged even after removing cases with RLS (Tables 1 and 2).

## DISCUSSION

This study showed that 37.4% of subjects with CLD suffered from poor sleep quality and nearly 20% of them reported insomnia. After excluding cases of RLS, poor sleep quality was reported in 30.18% and insomnia in 15.09% of subjects, which is higher than the prevalence in the general population^11,28^. Interestingly, sleep quality and insomnia were not associated with an abnormality in any of the laboratory parameters and severity of liver dysfunction, but with the presence of RLS and depression.

Earlier studies have shown that poor sleep quality has been reported by 69%-81% of subjects and insomnia by 42% in earlier studies^6,8,9^. Thus, the prevalence of poor sleep quality and insomnia were lesser in the present study compared to previous studies. These findings could be ascribed to multiple factors in previous studies. First, subjects included in these studies were older by ten years compared to the present study^6,8,9^. Second, the proportion of women participants was greater in the two studies than in the present study^6,8,9^. Third, a sizable number of subjects were on prescription opiate therapy and benzodiazepine therapy^6^. Fourth, complaints of poor sleep quality and insomnia could have manifested in proportion to the population prevalence of poor sleep quality and insomnia^29^. Fifth, aging, gender, opiate prescription, and withdrawal from benzodiazepines and stimulants consumption are known risk factors for poor sleep quality and insomnia^1,8^. These factors were observed in past studies^6,8,9^. Contrarily, the population included in the present study was dissimilar to earlier studies in being younger, having a predominance of males, and the absence of psychotropic medications or stimulants. Lastly, previous studies are silent regarding the presence of RLS in the study population, which could have inflated the proportion of poor sleep quality and insomnia^6,8,9^.

Unlike earlier studies, the present study failed to find any association between biochemical factors and sleep quality or insomnia. Insomnia arises out of dysfunctional neurocircuitry and it is difficult to describe the direct role of previously identified biochemical parameters, e.g., serum albumin and low hemoglobin in worsening sleep quality and insomnia^6,9^. However, as reported earlier, muscle cramps are painful and may induce sleep disturbances^6^. However, this parameter was not assessed during the present study, and hence, we cannot comment on it.

Previous studies reported that subjects with Child-Turcotte-Pugh grades A and/or B were at higher risk of having poor sleep quality or insomnia^6,8,9^. Though these effects disappeared during multivariate analysis in one study, but not in other^8,9^. Unlike previous studies, the present study did not find any association between the severity of CLD as measured by the Child-Turcotte-Pugh score and sleep-quality even in univariate analysis^6,9^. Even the MELD score was comparable between groups before and after removing subjects with RLS. Thus, at present, the role of severity of CLD is not well established as a risk for poor sleep quality and insomnia.

This study reported that RLS was a risk factor for insomnia and poor sleep quality. RLS is associated with sleep onset or sleep maintenance insomnia30. Similarly, subjects with RLS often have associated periodic limb movement during sleep, which may be associated with micro-arousals^30^. As discussed earlier, quality of sleep is dependent upon a combination of restorative sleep, continuity of sleep, and energy during the day^2^. Reduction in total sleep time due to delayed sleep onset or recurrent arousals, as seen in patients with RLS can evoke feelings of non-restorative sleep and low energy during the day, leading to complaints of poor quality sleep. This association is further strengthened by the fact that optimal treatment of RLS improves sleep quality^30^.

Like any other scientific investigation, the present study also had some methodological limitations. First, the sample size was small and was male-dominated. Second, the diagnosis of insomnia was based on a score of insomnia severity index along with clinical diagnosis, which did not differentiate between acute insomnia and insomnia disorder according to the diagnostic and statistical manual 5 edition^1^. Third, etiologically, the sample size was non-homogenous and most of the patients had decompensated disease. Fourth, besides RLS, other causes for poor sleep quality, e.g., obstructive sleep apnea and pruritus were not examined in the present study. Fifth, stringent exclusion criteria would interfere with generalizing the results. Lastly, polysomnography was not done to assess objective parameters of sleep in the present study.

In conclusion, insomnia and poor sleep quality are more prevalent among patients with CLD. Poor sleep quality was associated with insomnia, depression, and RLS. However, even after the removal of RLS, the association between poor sleep quality and insomnia, depression remained significant.
